# Inflamed Mind: Multiple Genetic Variants of *IL6* Influence Suicide Risk Phenotypes in Interaction With Early and Recent Adversities in a Linkage Disequilibrium-Based Clumping Analysis

**DOI:** 10.3389/fpsyt.2021.746206

**Published:** 2021-10-29

**Authors:** Janos Bokor, Sara Sutori, Dora Torok, Zsofia Gal, Nora Eszlari, Dorka Gyorik, Daniel Baksa, Peter Petschner, Gianluca Serafini, Maurizio Pompili, Ian M. Anderson, Bill Deakin, Gyorgy Bagdy, Gabriella Juhasz, Xenia Gonda

**Affiliations:** ^1^Department of Forensic and Insurance Medicine, Semmelweis University, Budapest, Hungary; ^2^Department of Pharmacodynamics, Faculty of Pharmacy, Semmelweis University, Budapest, Hungary; ^3^NAP-2-SE New Antidepressant Target Research Group, Hungarian Brain Research Program, Semmelweis University, Budapest, Hungary; ^4^SE-NAP-2 Genetic Brain Imaging Migraine Research Group, Semmelweis University, Budapest, Hungary; ^5^Bioinformatics Center, Institute for Chemical Research, Kyoto University, Uji, Japan; ^6^Department of Neuroscience, Rehabilitation, Ophthalmology, Genetics, Maternal, and Child Health, Section of Psychiatry, University of Genoa, Genoa, Italy; ^7^IRCCS Ospedale Policlinico San Martino, Genoa, Italy; ^8^Department of Neurosciences Mental Health and Sensory Organs, Suicide Prevention Center, Sant'Andrea Hospital, University of Rome, Rome, Italy; ^9^Neuroscience and Psychiatry Unit, Division of Neuroscience and Experimental Psychology, School of Biological Sciences, Faculty of Biological, Medical, and Human Sciences, The University of Manchester and Manchester Academic Health Sciences Centre, Manchester, United Kingdom; ^10^Department of Psychiatry and Psychotherapy, Semmelweis University, Budapest, Hungary

**Keywords:** *IL6*, inflammation, suicide, suicide attempt, suicide ideation, childhood adversities, recent stress, gene-environment interaction

## Abstract

**Background:** Understanding and predicting suicide remains a challenge, and a recent paradigm shift regarding the complex relationship between the immune system and the brain brought attention to the involvement of inflammation in neuropsychiatric conditions including suicide. Among cytokines, IL-6 has been most frequently implicated in suicide, yet only a few candidate gene studies and without considering the effect of stress investigated the role of *IL6* in suicidal behaviour. Our study aimed to investigate the association of *IL6* variation with a linkage disequilibrium-based clumping method in interaction with childhood adversities and recent stress on manifestations along the suicide spectrum.

**Methods:** One thousand seven hundred and sixty-two participants provided information on previous suicide attempts, current suicidal ideation, thoughts of death, and hopelessness, and were genotyped for 186 variants in *IL6*. Early childhood adversities were recorded with an instrument adapted from the Childhood Trauma Questionnaire, recent life events were registered using the List of Threatening Life Events. Following a 3-step quality control, logistic and linear regression models were run to explore the effect of genotype and gene-environment interactions on suicide phenotypes. All regression models were followed by a clumping process based on empirical estimates of linkage disequilibrium between clumps of intercorrelated SNPs. Interaction effects of distinct types of recent life events were also analysed.

**Results:** No clumps with significant main effects emerged, but we identified several clumps significantly interacting with childhood adversities on lifetime suicide attempts, current suicidal ideation, and current thoughts of death. We also identified clumps significantly interacting with recent negative life events on current suicidal ideation. We reported no clumps with significant effect on hopelessness either as a main effect or in interaction with childhood adversities or recent stress.

**Conclusion:** We identified variant clumps in *IL6* influencing suicidal behaviour, but only in interaction with childhood or recent adversities. Our results may bring us a step further in understanding the role of neuroinflammation and specifically of IL-6 in suicide, towards identifying novel biological markers of suicidal behaviour especially in those exposed to stressful experiences, and to fostering the adaptation of a new paradigm and identifying novel approaches and targets in the treatment of suicidal behaviour.

## Introduction

In spite of advances in neurobiology and neuropsychiatry, understanding and predicting suicide remains a challenge. Several clinical, psychosocial and socio-demographic risk factors and prognostic indicators for suicidal behaviour (1) have now been identified, however, with a low predictive value (2) and often even of a non-modifiable nature. Thus, more objective markers, especially biomarkers, and possibly reflecting modifiable processes, are imminently needed to improve prediction, risk screening and assessment, as well as to develop efficient methods for prevention and intervention. Furthermore, we need better insight into the neurobiological processes to offer a novel understanding of pathophysiology of suicide and identify new treatment targets and approaches (3).

While there has been a long-standing view that the brain is isolated from the peripheral immune system, recently a paradigm shift happened based on understanding of the multiple layers of immune surveillance in the central nervous system (4). Peripherally produced cytokines may cross the blood-brain barrier conveying signals to CNS, and with cytokines that are secreted locally by microglia, astrocytes and endothelial cells in the brain play a relevant role in the development and maintenance of brain function (5). While mental illnesses are not immunological disorders, the immune system potentially participates in subgroups of symptoms across multiple psychiatric disorders (4). Previous systematic reviews concluded that neuroinflammation may play a crucial role in the pathophysiology of suicidal behaviour including ideation, attempts and suicidal death. Still, more detailed knowledge of pathophysiological mechanisms underlying suicidal behaviour are needed including the possible mediators and moderators of inflammatory response that enhance vulnerability or resilience to suicide (6).

Proinflammatory states with higher levels of proinflammatory cytokines in blood, CSF and post-mortem brain are associated with different forms of suicidal behaviour and ideation (3, 7–14), suggesting that cytokine activation may impact suicidal behaviour in vulnerable individuals. Among cytokines, IL-6 is most frequently associated with suicidal behaviours (15), and assessment of IL-6 level is increasing in behavioural and psychosocial research due to its role in orchestrating inflammatory response and association with mental and physical health outcomes (16).

The majority, about 90% of suicidal behaviour occurs in the context of psychiatric disorders (17), but only a small minority, about 5% of psychiatric patients die by suicide, which suggests that beyond genetic and biochemical factors, environmental factors and stressors, as well as a dysfunction of the stress response system resulting in a maladaptive stress response also play a role in the emergence of suicide (18). The fact that the stress response is strongly associated with the immune system also supports that inflammation is one promising candidate for furthering our understanding of suicidal behaviours (3, 19, 20). Psychosocial stress is associated with upregulated gene expression and systemic inflammation markers, especially *IL6* (21). Thus, genetic variability in the expression of inflammatory markers in response to stress may contribute to increased vulnerability. In spite of this, only very few studies investigated the association of variation in *IL6* gene with suicidal behaviour. Furthermore, even those focused only on candidate variants, and did not consider the context of current or early stressors and adverse experiences.

As suicidal death is, fortunately, a relatively rare event, for clinical research other manifestations along the suicidal continuum are used as proxies, including suicidal ideation on non-lethal attempts which are strong predictors of the occurrence of subsequent completed suicide (22).

Thus, our present study aimed to investigate the impact of variation along the *IL6* gene with a linkage disequilibrium-based clumping method in interaction with early childhood adversities and recent negative life events on multiple manifestations of suicide including lifetime suicide attempts and current suicidal ideation in a larger European general population sample.

## Methods

### Sample

Volunteers aged 18–60 from Budapest and Greater Manchester were recruited to participate in the NewMood study (New Molecules in Mood Disorders, Sixth Framework Program of the European Union LHSM-CT-2004-503474) without receiving any compensation *via* advertisements, an online platform, and general practices. After providing written informed consent, participants received a saliva-based genetic sampling kit for genotyping and a questionnaire pack, consisting of a detailed background evaluation and multiple sub-units for in-depth phenotyping of several depression-related markers and endophenotypes (23, 24). Of the NewMood dataset, comprehensive mapping to enable exclusion of related individuals and those with incomplete relevant phenotypic and genotypic data yielded the present sample of 1,762 unrelated adults with self-reported European ethnic white origin, who were genotyped for the *IL6* gene.

Ethical approval was obtained from both local ethics committees (North Manchester Local Research Ethics Committee, Manchester, UK, and Scientific and Research Ethics Committee of the Medical Research Council, Budapest, Hungary). The study was carried out in accordance with the Declaration of Helsinki.

### Phenotyping

#### Phenotypes Related to the Suicide Spectrum

In the present study we focused on four suicide- and suicide risk-related phenotypes in order to grab a larger part of the suicidal behaviour spectrum, including previous suicide attempts, current suicidal ideation, and markers of current suicide risk including hopelessness and thoughts of death.

Previous lifetime suicide attempt (SUIC) was based on self-report providing a dichotomous variable. Current suicidal ideation (SI-BSI03) was evaluated using item 3, “Thoughts of ending your life” of the Brief Symptom Inventory (BSI) (25). Hopelessness (H-BSI18), a well-established independent risk factor of suicide was evaluated using BSI item 18 “Feeling hopeless about the future,” while thoughts of death (ToD-BSI21) were measured using BSI item 21 “Thoughts about death and dying.” These variables were scored on a scale from 0 to 4 (“Not at all” to “Extremely”) based on severity during the prior week to the assessment.

#### Measures of Environmental Stressors

To be able to evaluate gene-environment interactions contributing to the emergence of the measured suicidal behaviours, we assessed two types of environmental stressors, including childhood adversities (CHA) and recent negative life events (RLE). Childhood trauma and adversity (CHA) was assessed with an instrument derived from the Childhood Trauma Questionnaire (CTQ) (26) using the sum of four items on parental emotional abuse, physical abuse, and parental neglect, expanded by two more items on parental loss, as validated previously (24). Recent negative life events (RLE) occurring within 1 year prior to the assessment were recorded *via* the List of Threatening Experiences (27) encompassing four types of life events related to intimate relationships, financial difficulties, personal problems such as health-related or legal difficulties, and the social network (28).

### Genotyping

Participants provided buccal mucosa cells *via* a genetic sampling kit including a cytology brush (Cytobrush plus C0012, Durbin PLC) sent by mail to detect DNA. Extraction of genomic DNA was carried out according to the protocol of Freeman et al. (29). Genotyping was performed using Illumina CoreExom PsychChip. Genotyping was carried out in accordance with ISO 9001:2000 quality management requirements, and was blinded regarding phenotype.

Variant annotation was carried out based on the GRCh37/hg19 human assembly. For the purpose of phasing SHAPEIT was used to estimate additional haplotypes, followed by imputation of missing genotypes *via* IMPUTE2. This process yielded 186 SNPs on the *IL6* gene with boundaries extended by 10 kilobase (kb) pairs at both sides.

### Statistical Analyses

Genotyping provided a dataset including 1,762 individuals genotyped for 186 SNPs in the *IL6* gene (with boundaries extended by 10 kb) in the NewMood database. For SNPs we applied a 3-step quality control protocol including calculation of Hardy-Weinberg Equilibrium (HWE; >1 × 10^−5^), missingness rates (MF; <0.05), and minor allele frequencies (MAF; >0.01) along the criteria suggested by Coleman et al. (30). For quality control purposes and main effect/interaction models Plink v1 0.9 was used. 86 SNPs survived quality control and were entered into analyses.

Logistic and linear regression models were applied to explore the effect of genotype and gene-environment interactions on lifetime suicide attempts (SUIC) as a dichotomous variable and on current suicidal ideation (SI-BSI03), hopelessness (H-BSI18) and current thoughts of death (ToD-BSI21) as continuous outcome variables, respectively. First, main effects of genetic variation on all phenotypes were analysed (Supplementary Table 1), followed by gene-environment interaction models with early childhood adversities (CHA) in case of suicide attempts (SUIC), current suicidal ideation (SI-BSI03), hopelessness (H-BSI18) and current thoughts of death (ToD-BSI21) (Supplementary Table 2); and with recent negative life events (RLE) in case of current suicidal ideation (SI-BSI03), hopelessness (H-BSI18) and current thoughts of death (ToD-BSI21) (Supplementary Table 3).

Next, all regression models, including main effect and interaction, were followed up by a clumping process using the CLUMP function in Plink. Clumping is a statistical method for yielding clumps of intercorrelated SNPs based on empirical estimates of their linkage disequilibrium (LD), stepping beyond independent significance levels, identifying connected SNPs and their index or top SNP (the one with the highest significance). The four parameters used for clumping were as follows: (1) maximum *p*-value of the clump's top SNP was 0.001; (2) maximum *p*-value for secondary SNPs was 0.05; (3) minimum LD *R*^2^ was 0.5; and (4) physical distance threshold was 250 kilobases.

As secondary analyses, we also analysed the interaction effects of different types of recent life events including financial difficulties (RLE-financial), personal problems (RLE-personal), intimate relationship problems (RLE-relationship), and social network disturbances (RLE-social) (28) on current indicators of suicide risk. Intercorrelations between the subscales have also been previously reported, and were either negligibly weak or non-significant (31).

All analyses were run according to additive, dominant, and recessive models. In all statistical models, population (Manchester or Budapest), gender and age were entered as covariates. In addition, in the case of gene-environment interaction models, the main effects of both the SNP and the stressors (CHA/RLE) were also included as covariates.

**Figure 1:**
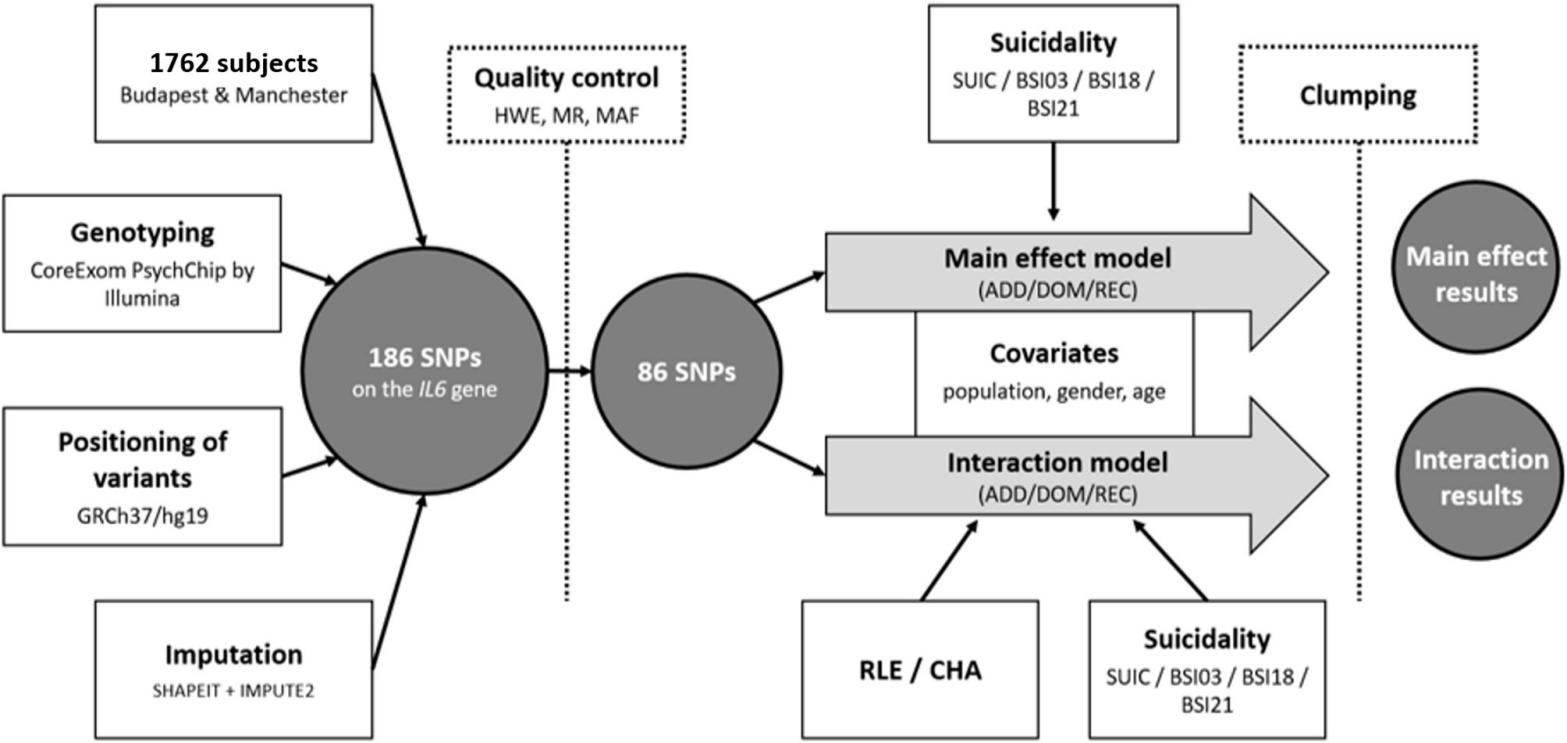
Sample, methodology and statistical analyses used to investigate effect of *IL6* gene variations on lifetime suicide attempts (SUIC) and suicide items of the Brief Symptom Inventory (BSI) both in main effect models and in interaction with proximal (recent negative life events) and distal (childhood adversities) stressors. SNP, Single Nucleotide Polymorphism; SUIC, lifetime suicide attempts; BSI, Brief Symptom Inventory; SI-BSI03, current suicidal ideation (“Thoughts of ending your life”); H-BSI18, current hopelessness (“Feeling hopeless about the future”); ToD-BSI21, current thoughts of death (“Thoughts about death and dying”); RLE, Recent negative Life Events; CHA, Childhood Adversity; HWE, Hardy-Weinberg Equilibrium; MR, Missing Rate; MAF, Minor Allele Frequency; ADD, additive model; DOM, dominant model; REC, recessive model.

Nominal significance threshold was set at *p* < 0.05. Bonferroni-method was used to correct for multiple comparisons. Assuming 33 models [in main effect: 4 outcome variables (SUIC/SI-BSI03/H-BSI18/ToD-BSI21), × 3 genetic models (additive/dominant/recessive) = 12 models; in interaction: 1 outcome variable (SUIC) × 1 environmental variable (CHA) × 3 genetic models (additive/dominant/recessive) = 3; and 3 outcome variables (SI-BSI03/H-BSI18/ToD-BSI21) × 2 environmental variables (CHA/RLE) × 3 genetic models (additive/dominant/recessive) = 18 models] the corrected significance threshold was set at *p* = 0.0015. For the secondary analyses, assuming 36 models [3 outcome variables (SI-BSI03/H-BSI18/ToD-BSI21) × 4 environmental variables (RLE intimate/financial/personal/social) × 3 genetic models (additive/dominant/recessive) = 36 models], significance threshold was set at *p* = 0.0014. The main steps of genotyping and the statistical analyses are illustrated in Figure 1.

In the above analyses, Plink v1.90 was used to calculate missingness rate (MR; <0.05), Hardy-Weinberg equilibrium (HWE; >1 × 10^−5^) and minor allele frequency (MAF; >0.01) as part of quality control steps prior to the analyses; for clumping; and for building linear and logistic regression models to test for main and interaction effects of genetic variation in the *IL6* gene. Analyses were supported by scripts individually written in R 3.0.2 (R Core Team, 2013). R was also used to illustrate the effects of significant findings (version 4.0.3 with the ggplot2 package). Descriptive statistics were run using IBM SPSS Statistics 25.

All data used in the study were openly shared and are available in the Figshare depository at https://figshare.com/s/6d0f6f781466c78a7c0a.

## Results

### Description of the Sample

Descriptive statistics of the analysed sample are shown in Table 1.

**Table 1:** Characteristics of the sample.

	***N***		**%**	
Total	1,762			
**Gender**
Male	504		28.60%	
Female	1,258		71.40%	
Lifetime suicide attempt	208		11.80%	
	**Minimum**	**Maximum**	**Mean**	**SD**
Age	18	60	32.56	10.47
SI-BSI03 Current suicidal ideation	0	4	0.34	0.86
H-BSI18 Hopelessness	0	4	0.95	1.31
ToD-BSI21 Current thoughts of death	0	4	0.67	1.16
RLE	0	8	1.21	1.27
CHA	0	16	3.28	3.34

*BSI, Brief Symptom Inventory; RLE, recent negative life events; CHA, childhood adversity; SD, standard deviation*.

### Main Effect of *IL6* Variation on Lifetime Suicide Attempts, Current Suicidal Ideation, Current Hopelessness, and Current Thoughts of Death

Logistic regression models with SUIC (for lifetime suicide attempts) and linear regression models with SI-BSI03, H-BSI18, and ToD-BSI21 (for suicidal ideation and other markers of current suicidal risk) identified several individual SNPs reaching nominal significance threshold, however, as all *p*-values exceeded the maximum threshold specified for clumping (*p* = 0.001), clumps could not be identified.

### Effects of Early Childhood Adversities in Interaction With *IL6* Variation on Lifetime Suicide Attempts, Current Suicidal Ideation, Current Hopelessness, and Current Thoughts of Death

**Figure 2:**
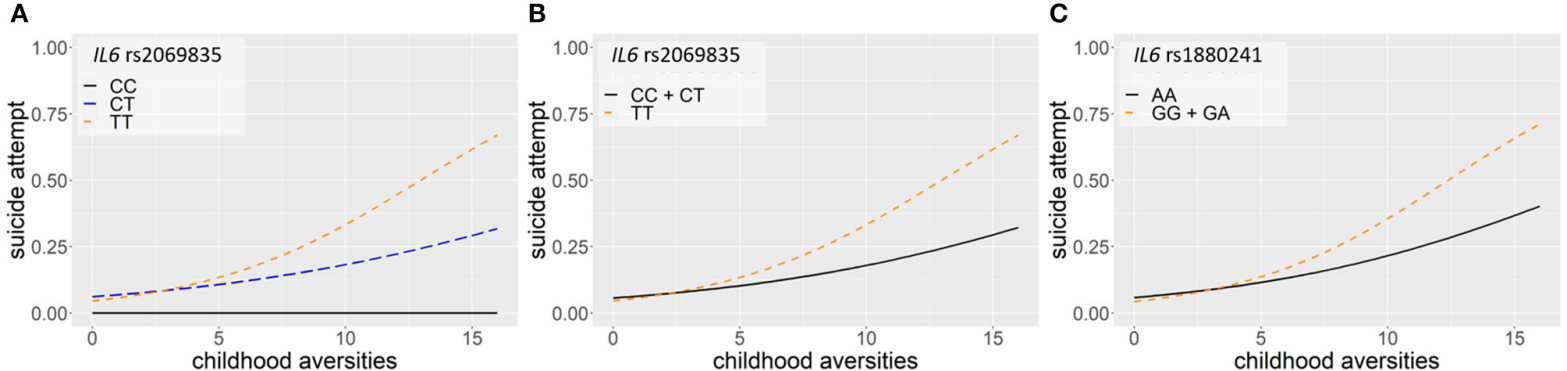
Logistic regression models on the interaction between *IL6* and childhood adversities on history of lifetime suicide attempts yielded three significant clumps (one for additive and two for dominant models), surviving correction for multiple testing. In the additive model, a clump containing three SNPs emerged with rs2069835 as the top SNP and the minor C allele as a protective allele (*p* = 0.0004) **(A)**. In the dominant model, a clump containing 4 SNPs with rs 2069835 as the top SNP and the minor C allele as a protective allele (*p* = 0.0004) **(B)**, and another clump containing 3 SNPs, with rs1880241 as the top SNP and the minor G allele as a risk allele (*p* = 7.08 × 10^−5^) **(C)** was identified. The figure was generated for illustration purposes using R.

Logistic regression models on the interaction between *IL6* and childhood adversities on lifetime suicide attempts yielded three significant clumps (one for additive and two for dominant models), surviving correction for multiple testing (Table 2; Figure 2). In the additive model, a clump containing three SNPs emerged with rs2069835 as the top SNP and the minor C allele as a protective allele (*p* = 0.0004; Figure 2A). In the dominant models, one identified clump contained four SNPs with rs2069835 as the top SNP and the minor C allele as a protective allele (*p* = 0.0004; Figure 2B), while the other contained 3 SNPs, with rs1880241 as the top SNP and the minor G allele as a risk allele (*p* = 7.08 × 10^−5^; Figure 2C).

**Table 2:** Interaction effects between *IL6* variation and childhood adversities on lifetime suicide attempts.

***IL6 × * CHA: lifetime suicide attempts**						
**SNP**	**OR**	**SE**	**95%CI**	**Minor allele**	**Risk/protective**	***p***
**ADDITIVE MODEL**						
**rs2069835**	0.47	0.21	0.31–0.72	C	Protective	0.0004
rs13438415	0.52	0.21	0.35–0.79	A	Protective	0.0020
rs2069824	0.53	0.22	0.35–0.81	C	Protective	0.0032
**DOMINANT MODEL**						
**rs1880241**	2.04	0.18	1.44–2.90	G	Risk	0.0001
rs62449494	1.94	0.18	1.38–2.74	G	Risk	0.0002
rs7805828	2.01	0.18	1.42–2.83	A	Risk	0.0001
**rs2069835**	0.46	0.22	0.30–0.70	C	Protective	0.0003
rs7801617	0.64	0.22	0.41–0.99	A	Protective	0.0439
rs13438415	0.51	0.21	0.33–0.77	A	Protective	0.0014
rs2069824	0.51	0.22	0.34–0.79	C	Protective	0.0022

*Top SNP of each clump are marked in bold, member SNPs in each clump are listed under the top SNP. P-values surviving corrections for multiple testing are shown. CHA, childhood adversity; OR, odds ratio; SE, standard error; 95%CI, 95% confidence interval*.

**Figure 3:**
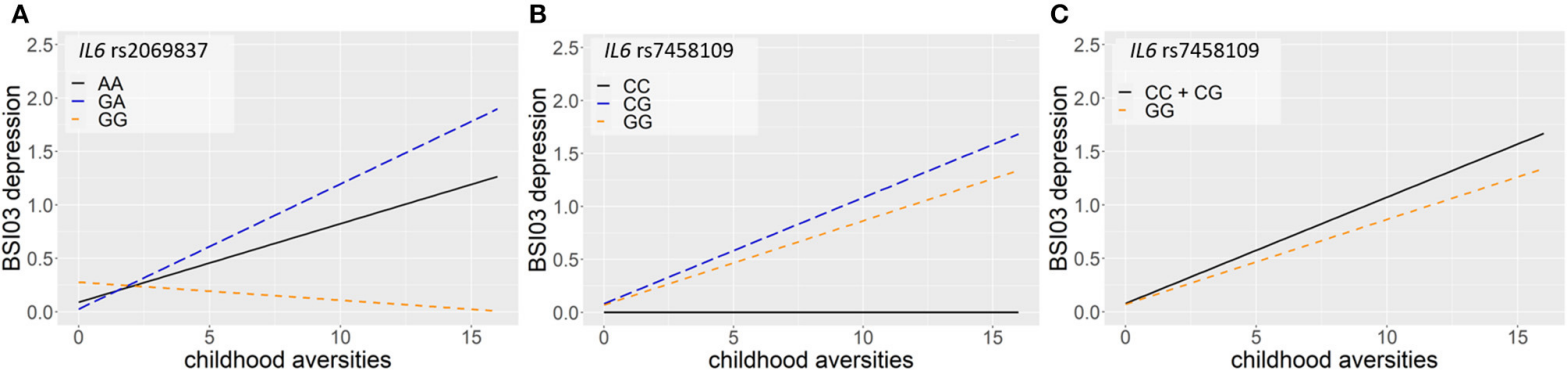
Linear regression models on the interaction between *IL6* and childhood adversities on current suicidal ideation (SI-BSI03) yielded three significant clumps, consisting of one SNP each. In the additive model, one identified clump contained rs2069837 with the minor allele G as a risk allele (*p* = 0.0006) **(A)**. Furthermore, one clump containing also one SNP, rs7458109, was significant in both additive and dominant models, with the minor C as a risk allele (*p* = 0.0006 and *p* = 0.0004 for additive and dominant models, respectively) **(B,C)**. The figure was generated for illustration purposes using R.

Linear regression models on the interaction between *IL6* and childhood adversities on current suicidal ideation (SI-BSI03) yielded three significant clumps (two for additive and one for dominant models) all consisting of one SNP each, surviving correction for multiple testing (Table 3; Figure 3). In the additive model, one identified clump contained rs2069837 with the minor allele G as a risk allele (*p* = 0.0006; Figure 3A). Furthermore, one clump containing only one SNP, rs7458109, was significant in both additive and dominant models, with the minor C as a risk allele (*p* = 0.0006 and *p* = 0.0004 for additive and dominant models, respectively) (Figures 3B,C).

**Table 3:** Interaction effects between *IL6* variation and childhood adversities on current suicidal ideation.

***IL6 × * CHA: current suicide risk**						
**SNP**	**Beta**	**SE**	**95%CI**	**Minor allele**	**Risk/protective**	***p***
**C****urrent suicidal ideation** **(SI-BSI03)**						
**ADDITIVE MODEL**						
**rs7458109**	0.48	0.14	0.21–0.76	C	Risk	0.0006
**rs2069837**	0.30	0.09	0.13–0.48	G	Risk	0.0006
**DOMINANT MODEL**						
**rs7458109**	0.51	0.14	0.23–0.79	C	Risk	0.0004

*Top SNP of each clump are marked in bold, other SNPs in each clump are listed under the top SNP. P-values surviving corrections for multiple testing are shown. SE, standard error; 95%CI, 95% confidence interval*.

Linear regression models on the interaction between *IL6* and childhood adversities on current hopelessness (H-BSI18) yielded no significant clumps.

**Figure 4:**
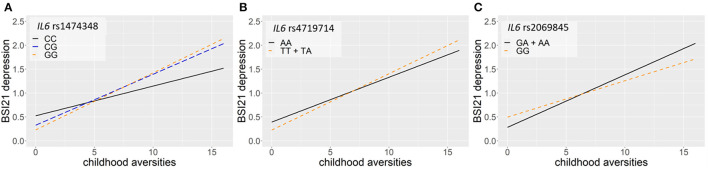
Linear regression models on the interaction between *IL6* and childhood adversities on current thoughts of death (ToD-BSI21) yielded three significant clumps. In case of the additive model, a clump containing 24 SNPs with rs1474348 as top SNP and the minor C allele as a protective allele emerged (*p* = 0.0005) **(A)**. In case of the dominant model, a clump containing 5 SNPs with top SNP rs4719714 and the minor T allele as a risk allele was found (*p* = 0.0007) **(B)**. In the recessive model, a clump containing 19 SNPs with rs2069845 as top SNP and the minor G allele as a protective allele was identified (*p* = 0.0002) **(C)**. The figure was generated for illustration purposes using R.

Linear regression models on the interaction between *IL6* and childhood adversities on current thoughts of death (ToD-BSI21) yielded three significant clumps (one for additive, dominant and recessive models) surviving correction for multiple testing (Table 4; Figure 4). In case of the additive model, a clump containing 24 SNPs with rs1474348 as top SNP and the minor C allele as a protective allele emerged (*p* = 0.0005; Figure 4A). In case of the dominant model, a clump containing 5 SNPs with top SNP rs4719714 and the minor T allele as a risk allele was found (*p* = 0.0007; Figure 4B). In the recessive model, a clump containing 19 SNPs with rs2069845 as top SNP and the minor G allele as a protective allele was identified (*p* = 0.0002; Figure 4C).

**Table 4:** Interaction effects between *IL6* variation and childhood adversities on current thoughts of death.

***IL6*** **× ** **CHA: current suicide risk**						
**SNP**	**Beta**	**SE**	**95%CI**	**Minor allele**	**Risk/protective**	***p***
**C****urrent thoughts of death** **(ToD-BSI21)**				
**ADDITIVE MODEL**						
**rs1474348**	−0.15	0.04	−0.24 to −0.07	C	Protective	0.0005
rs6461662	−0.09	0.04	−0.18 to −0.01	G	Protective	0.0273
rs6963591	−0.09	0.04	−0.18 to −0.01	A	Protective	0.0315
rs6963866	−0.09	0.04	−0.18 to −0.01	C	Protective	0.0290
rs1546762	−0.09	0.04	−0.18 to −0.01	C	Protective	0.0314
rs1546763	−0.09	0.04	−0.18 to −0.01	G	Protective	0.0314
rs4719713	−0.09	0.04	−0.18 to −0.01	G	Protective	0.0302
rs1880241	0.09	0.04	0.01 to 0.17	G	Risk	0.0240
rs1880242	−0.10	0.04	−0.18 to −0.01	G	Protective	0.0213
rs2002792	0.11	0.04	0.02 to 0.19	G	Risk	0.0142
rs7802307	−0.15	0.04	−0.23 to −0.06	T	Protective	0.0009
rs1800795	−0.15	0.04	−0.24 to −0.07	C	Protective	0.0006
rs2069832	−0.15	0.04	−0.24 to −0.07	A	Protective	0.0006
rs1474347	−0.15	0.04	−0.24 to −0.07	C	Protective	0.0005
rs1554606	−0.15	0.04	−0.23 to −0.06	T	Protective	0.0006
rs2069845	−0.15	0.04	−0.23 to −0.06	G	Protective	0.0005
rs7787893	−0.13	0.04	−0.22 to −0.05	A	Protective	0.0019
rs35436671	−0.13	0.04	−0.22 to −0.05	G	Protective	0.0023
rs12700390	−0.13	0.04	−0.22 to −0.04	G	Protective	0.0030
rs12700391	−0.13	0.04	−0.22 to −0.04	C	Protective	0.0030
rs7781534	−0.13	0.04	−0.22 to −0.04	A	Protective	0.0031
rs4722168	−0.13	0.04	−0.22 to −0.04	G	Protective	0.0033
rs1581497	−0.13	0.04	−0.22 to −0.05	C	Protective	0.0028
rs1829927	−0.13	0.04	−0.22 to −0.04	G	Protective	0.0031
**DOMINANT MODEL**						
**rs4719714**	0.22	0.06	0.09 to 0.34	T	Risk	0.0007
rs56728381	0.21	0.06	0.09 to 0.34	C	Risk	0.0008
rs73683966	0.20	0.06	0.08 to 0.33	G	Risk	0.0016
rs73683967	0.20	0.06	0.08 to 0.33	G	Risk	0.0013
rs10499563	0.21	0.06	0.09 to 0.34	G	Risk	0.0008
**RECESSIVE MODEL**						
**rs2069845**	−0.27	0.07	−0.42 to −0.13	G	Protective	0.0002
rs7802307	−0.25	0.08	−0.40 to −0.10	T	Protective	0.0012
rs1800795	−0.27	0.08	−0.42 to −0.12	C	Protective	0.0005
rs2069832	−0.26	0.08	−0.41 to −0.11	A	Protective	0.0007
rs1474348	−0.26	0.08	−0.41 to −0.11	C	Protective	0.0006
rs1474347	−0.26	0.08	−0.41 to −0.11	C	Protective	0.0006
rs1554606	−0.27	0.07	−0.42 to −0.13	T	Protective	0.0002
rs7787893	−0.23	0.08	−0.37 to −0.08	A	Protective	0.0028
rs35436671	−0.23	0.08	−0.38 to −0.07	G	Protective	0.0041
rs12700390	−0.22	0.08	−0.38 to −0.06	G	Protective	0.0056
rs12700391	−0.22	0.08	−0.38 to −0.06	C	Protective	0.0056
rs7781534	−0.22	0.08	−0.38 to −0.07	A	Protective	0.0054
rs4722168	−0.22	0.08	−0.38 to −0.07	G	Protective	0.0053
rs1581497	−0.22	0.08	−0.38 to −0.07	C	Protective	0.0049
rs1829927	−0.22	0.08	−0.38 to −0.07	G	Protective	0.0054

*Top SNP of each clump are marked in bold, other SNPs in each clump are listed under the top SNP. P-values surviving corrections for multiple testing are shown. SE, standard error; 95%CI, 95% confidence interval*.

### Effects of Recent Negative Life Events in Interaction With *IL6* Variation on Current Suicidal Ideation, Current Hopelessness, and Current Thoughts of Death

**Figure 5:**
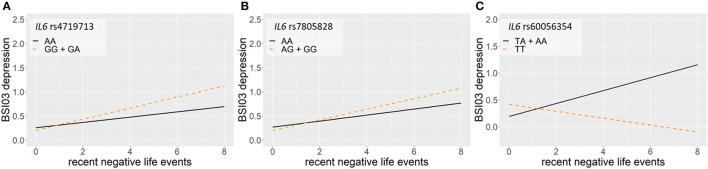
Linear regression models on the interaction between *IL6* and recent negative life events (RLE) on current suicidal ideation (SI-BSI03) yielded three significant clumps. In the dominant model, a clump containing seven SNPs with top SNP rs4719713 and the minor G allele as a risk allele was identified (*p* = 0.0003) **(A)**. In the recessive model, one identified clump contained 4 SNPs with top SNP rs7805828 and the minor A allele as a protective allele (9 = 7.39 × 10^−5^) **(B)**; while the other clump contained six SNPs with top SNP rs60056354 and the minor T allele as a protective allele (*p* = 0.0007) **(C)**. The figure was generated for illustration purposes using R.

Linear regression models on the interaction between *IL6* and recent negative life events (RLE) on current suicidal ideation (SI-BSI03) yielded three significant clumps (one for dominant and two for recessive models) surviving correction for multiple testing (Table 5; Figure 5). In the dominant model, a clump containing seven SNPs with top SNP rs4719713 and the minor G allele as a risk allele was identified (*p* = 0.0003; Figure 5A). In the recessive model, one identified clump contained 4 SNPs with top SNP rs7805828 and the minor A allele as a protective allele (*p* = 7.39 × 10^−5^; Figure 5B); while the other clump contained six SNPs with top SNP rs60056354 and the minor T allele as a protective allele (*p* = 0.0007; Figure 5C).

**Table 5:** Interaction effects of *IL6* variation and recent negative life events on current markers of suicidality.

***IL6*** **× ** **RLE: current suicide risk**						
**SNP**	**Beta**	**SE**	**95%CI**	**Minor allele**	**Risk/protective**	***p***
**C** **urrent suicidal ideation (** **SI-BSI03)**						
**DOMINANT MODEL**						
**rs4719713**	0.27	0.07	0.12 to 0.42	G	Risk	0.0003
rs6461662	0.27	0.07	0.12 to 0.42	G	Risk	0.0003
rs6963591	0.27	0.07	0.12 to 0.42	A	Risk	0.0003
rs6963866	0.27	0.07	0.12 to 0.41	C	Risk	0.0004
rs1546762	0.27	0.07	0.12 to 0.42	C	Risk	0.0003
rs1546763	0.27	0.07	0.12 to 0.42	G	Risk	0.0003
rs1880242	0.27	0.07	0.12 to 0.42	G	Risk	0.0003
**RECESSIVE MODEL**						
**rs7805828**	−0.32	0.08	−0.48 to −0.16	A	Protective	7.39E-05
rs62449494	−0.31	0.08	−0.47 to −0.15	G	Protective	0.0002
rs1880241	−0.19	0.08	−0.34 to −0.04	G	Protective	0.0146
rs2002792	−0.29	0.08	−0.44 to −0.14	G	Protective	0.0002
**rs60056354**	−0.38	0.11	−0.61 to −0.16	T	Protective	0.0007
rs367801961	−0.38	0.11	−0.61 to −0.16	A	Protective	0.0007
rs35610689	−0.39	0.12	−0.63 to −0.16	G	Protective	0.0018
rs34880821	−0.39	0.12	−0.63 to −0.16	A	Protective	0.0012
rs66543531	−0.40	0.12	−0.63 to −0.16	T	Protective	0.0011
rs6954667	−0.39	0.12	−0.62 to −0.16	A	Protective	0.0008

*Top SNP of each clump are marked in bold, other SNPs in each clump are listed under the top SNP. P-values surviving corrections for multiple testing are shown. SE, standard error; 95%CI, 95% confidence interval*.

No significant clumps emerged in linear regression models on the interaction between *IL6* and recent negative life events (RLE) either on current hopelessness (H-BSI18), or current thoughts of death (ToD-BSI21).

### Effects of Distinct Types of Recent Negative Life Events in Interaction With *IL6* Variation on Current Suicidal Ideation, Current Hopelessness, and Current Thoughts of Death

Linear regression models on the interaction between *IL6* and recent negative life events related to financial problems (RLE-financialC2) on current suicidal ideation (SI-BSI03) yielded two significant clumps surviving correction for multiple testing, one of which was significant in both additive and dominant models. In the additive and dominant models, a clump containing three SNPs with top SNP exm609179 and the minor A allele as a risk allele was identified (*p* = 0.0002), while in the recessive model a clump with 15 SNPs including top SNP rs1554606 and the minor T allele as a protective was found (*p* = 0.0002). In interaction with life events related to personal problems (RLE-personalC3) linear regression identified one clump in the *IL6* gene which survived correction for multiple testing in both additive and dominant models, containing 6 SNPs including top SNP rs34328912 with the minor C allele as a risk allele (*p* = 0.0003). Life events related to intimate relationships (RLE-intimateC1) or social network (RLE-socialC4) did not interact with *IL6* variation on current suicidal ideation.

On the other hand, recent intimate relationship-related life events and social network-related life events interacted with *IL6* variation on current thoughts of death (ToD-BSI21). In case of recent intimate relationship life events (RLE-intimateC1) two interacting clumps surviving significance for multiple testing were identified. The clump included 16 SNPs in the additive and 14 SNPs in the dominant model; top SNP was rs7802307 for both models with the minor T allele as a protective allele (*p* = 0.0008 and *p* = 0.0009 for additive and dominant models, respectively). There was also a significant interaction between *IL6* variation and recent life events related to the social network (RLE-socialC4) according to the recessive model surviving correction for multiple testing yielding one clump including 2 SNPs with top SNP rs7802307 and the minor T allele as a protective allele (*p* = 0.0005). In case of current thoughts of death (ToD-BSI21), no significant interaction effects emerged in case of the other two types of life events, financial problems (RLE-financialC2) or personal problems (RLE-personalC3).

In case of hopelessness (H-BSI18), no significant interaction effects were found with any recent life event subtypes.

### *In silico* Characterisation and Functional Prediction of Identified Top IL6 SNPs as Well as SNPs in the Clumps Showing a Significant Effect on Suicidal Behaviours

**Figure 6:**
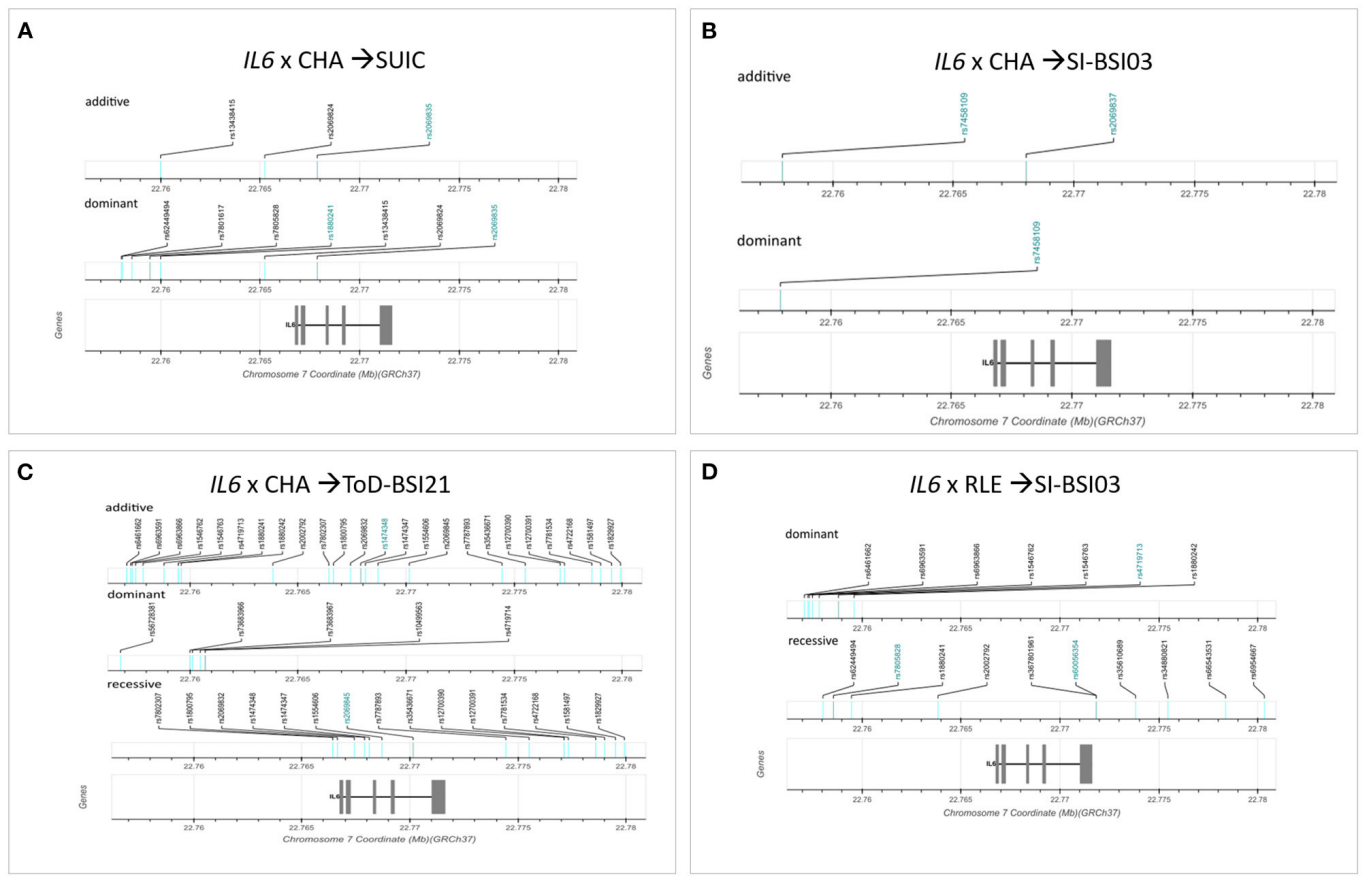
Genomic location of significant clumps of SNPs in the *IL6* gene influencing suicidal phenotypes in interaction with different types of stress. Logistic regression models on the interaction between *IL6* and childhood adversities on lifetime suicide attempts yielded three significant clumps (one for additive and two for dominant models) **(A)**. Linear regression models on the interaction between *IL6* and childhood adversities on current suicidal ideation (SI-BSI03) yielded three significant clumps (two for additive and one for dominant models) all consisting of one SNP each **(B)**. Linear regression models on the interaction between *IL6* and childhood adversities on current thoughts of death (ToD-BSI21) yielded three significant clumps **(C)**. Linear regression models on the interaction between *IL6* and recent negative life events (RLE) on current suicidal ideation (SI-BSI03) yielded three significant clumps **(D)**. Top SNPs are marked with blue for each clump. The exons are marked with grey rectangles on the gene. Figures are based on diagrams generated by LDlink, LDmatrix Tool (https://ldlink.nci.nih.gov/?tab=ldmatrix).

Genomic location of significant SNPs and top SNPs identified in the clumping procedure are shown in Figure 6. Figures are based on diagrams generated by LDlink, LDmatrix Tool (https://ldlink.nci.nih.gov/?tab=ldmatrix).

To detect the functional effect of the top significant SNPs, we utilised FuncPred (https://snpinfo.niehs.nih.gov), and SNPNexus (https://www.snp-nexus.org/v4/guide/#cons), including DeepSea to predict significance of predicted chromatin effect and evolutionary conservation; FunSeq2 to calculate a non-coding score for the identified variants; GWAVA to calculate functional probability, and Genomic Evolutionary Rate Profiling. Results are shown in Supplementary Tables 4–12 separately for all analyses where significant clumps were identified.

## Discussion

Our study investigated the effect of multiple variants in the *IL6* gene with a linkage disequilibrium-based clumping method on different manifestations along the suicide spectrum, including lifetime suicide attempts as well as current suicide ideation and markers of current risk such as thoughts of death and hopelessness. We identified no clumps of variants with a main effect on any of the investigated phenotypes. However, significant effects of several clumps of SNPs were identified in interaction with environmental stressors in several models. In case of lifetime suicide attempts, in interaction with early childhood adversities we identified 3 clumps, one in an additive and two in dominant models, with rs206985 emerging as top SNP in both models. In case of current markers of suicide risk, in interaction with childhood adversities we identified 3 clumps influencing current suicidal ideation (with rs7458109 as top SNP in both additive and dominant models) and three clumps influencing current thoughts of death. In case of current ideation we also identified 3 clumps of variants interacting with recent negative life events, with top SNP rs4719713. Our results suggest that *IL6* variation influences suicidal behaviour and risk but only in interaction with environmental stress. The validity of our findings is further supported by the fact that while we focused on a single gene, we investigated the combined effects of all 186 SNPs of *IL6* available in the NewMood genetic database clumped based on linkage disequilibrium, but without prior theoretical selection of only a few candidate variants.

### The Effect of IL-6 on Brain and Behaviour

Cytokines are a heterogeneous group of regulatory polypeptides, released by immunocompetent cells such as lymphocytes and macrophages, playing a role in host defence and repair processes of tissues. and besides being able to cross the blood-brain barrier by active transport, are also produced in the brain by neurons, astrocytes and activated microglial cells, thus providing a direct link with the immune system (32). Cytokines are increasingly recognised as messenger molecules, that modulate neuroendocrine functions, participate in neuroinflammatory and neurodegenerative processes, and have also been implicated in neurodevelopmental disorders and in the neurobiology stress-related conditions such as mood disorders or suicide (6, 15, 33, 34). Proinflammatory cytokines IL-6, IL-1, and TNF-alpha appear to be the most relevant considering their actions in the brain (34), and one of their most remarkable and relevant characteristics is their pleiotropy to bind different cell target types (6).

IL-6 is a pleiotropic cytokine mediator of immune reactions with functions including production of acute phase proteins in liver, B-lymphocyte proliferation and differentiation, osteoclast activation, and fever induction in brain. There are two types of IL-6 signalling: the anti-inflammatory or classical pathway, and the proinflammatory pathway or transsignalling way. IL-6 binds to membrane-bound IL-6R in the classical pathway, and to soluble IL-6R in the proinflammatory pathway. The proinflammatory pathway is used by various cell types within the brain (34). IL-6 can directly bind to receptors on neurons in the central nervous system and has been shown to directly impact generation of action potentials, which may influence changes in emotion and behaviour including increased risk for suicidal intent and behaviour (35).

Cytokines including IL-6 contribute to changes in the brain and behaviour *via* several mechanisms. Cytokine receptors, including IL-6 receptors can be found in specific brain regions and can thus have a direct effect on neuronal function and induce specific effects on behaviour and emotion; for example, IL-6 receptors are located on serotonin neurons in the medulla oblongata, hypothalamus, hippocampus, cerebellum, and prefrontal cortex. Cytokines also modulate concentration and function of monoamine neurotransmitters and especially serotonin and metabolites in various brain regions implicated in the pathophysiology of suicide including the prefrontal cortex, hippocampus and amygdala (5, 14). Cytokines also induce changes in the function of the HPA-axis, the other major system implicated in suicidal behaviour (14). Furthermore, inflammation and increased cytokine levels are also associated with activation of the kynurenine pathway (36) with downstream production of metabolites with effects on glutamate neurotransmission (37). Ultimately, disruption of regulatory corticostriatal circuits as a consequence of increased cytokine levels lead to abnormalities in executive function, emotion regulation, hopelessness and impulsivity contributing to suicide vulnerability (38). However, given their role in the central nervous system, cytokines including IL-6 quite likely exert a profound influence *via* their effect on foetal and later neurodevelopment (39).

### The Effect of *IL6* Gene on Suicidal Behaviour

Genetic component of suicidal behaviour is estimated at 43% (40) with 30–50% for a broader suicidality phenotype (41). In a previous GWAS, strongest candidate genes of suicidal behaviour were linked to inflammatory response (42), and recent studies identifying top ranking molecular markers predicting suicidal ideation lead to 8 genes including *IL6* (besides *SAT1, SKA2, SLC4A4, KIF2C, MBP, JUN*, and *KLHDC3*) (16, 43, 44). The imbalance between anti- and proinflammatory cytokines in suicidal behaviour also suggests that cytokine genes may be putative contributors of suicide risk (45), with heritability for IL-6 levels estimated in twin studies ranging between 0.25 and 0.26 when taking into account confounding factors such as BMI (46).

*IL6* gene in humans is mapped to region p15-21 on chromosome 7. It includes four introns, five exons, encoding a precursor protein with a total length of 212 amino acids, yielding a 186-amino acid mature segment and a 28-amino acid signal sequence (34). A very recent study using an SNP-based correlation and Mendelian randomisation approach showed a consistent association between increased IL-6 signalling using different genetic proxies and suicidality (47).

In spite of its suspected role in suicidal behaviour, only a few studies directly investigated genetic variation in *IL6* in association with suicide. One study assessed two variants in suicide attempters, completers and controls, selected based on previous studies showing their role in *IL6* expression regulation (48, 49). While no difference was found in rs2069845 allele frequencies in the three groups, the C allele of promoter-based rs1800795, possibly influencing histone acetylation and transcription factor binding and associated with higher IL-6 levels (48, 49), was more common in completed than attempted suicide, and was also associated with the lethality of suicide attempts (45) suggesting that higher IL-6 levels may mediate this association. Interestingly, in a previous study the G allele of rs1800795 was found to be neuroprotective considering its role on hippocampal volume in healthy subjects (50). Considering that smaller hippocampal volume predicts suicide attempts in depressed patients (51), this association may account for one of the effects of *IL6* gene on suicide risk.

Remarkably, both of the candidate variants rs2069845 and rs1800795 investigated by the study of Eftekharian et al. emerged as members of significant clumps in our present analyses, which, in contrast, investigated all 186 SNPs available in the NewMood database without hypothesis-based variant pre-selection. However, in our analyses the mentioned variants did not influence suicidal behaviours as a main effect, but only in interaction with environmental events, and rs1800795 showed an opposite effect, as in our study the C allele appeared as a protective variant.

### Effects of *IL6* Variation on Suicidal Behaviour in Interaction With Environmental Stress

Our finding that effect of *IL6* variation was only observable in those exposed to some forms of environmental influences, either early traumas or recent negative life events, falls in line with previous observations that in several psychiatric conditions, including depression (31, 52–55), genetic factors do not directly influence manifestations, but rather exert their effects *via* influencing sensitivity towards environmental factors, and it seems to be the case also for both suicide and inflammation.

Stress has a well-known impact on neuropsychiatric disease which may in part be mediated *via* immunological mechanisms especially in the case of mood and anxiety disorders (4). Exposure to stressful life events and social stressors such as social defeat, loss, separation or rejection may cause impairments in various aspects of immune function, including stress-related changes in cytokine levels and function, and proinflammatory cytokine upregulation in both human and animal models (56). Inflammation increases threat-related neural sensitivity to negative social experiences to enhance sensitivity to threats to well-being or safety in order to avoid them, and also enhances reward-related neural sensitivity to positive social experiences to increase approach-related motivations towards others who might provide support and care (56). Psychological stress in clinical studies leads to elevated inflammation (57), with IL-6 levels up to four times higher in chronic stress compared to no stress (34, 58–61). Acute stress (62) and life-event related stress (63) also correlates with increased blood IL-6 levels (34).

Genetic variability likely interacts with environmental stressors leading to increased inflammatory markers in genetically susceptible individuals (14). However, only a few studies looked at the GxE effect in the background of increased IL-6 levels in the face of stress, and no previous studies investigated the effect of *IL6* variation on suicidal manifestations in a GxE approach. In one study, older adults losing a spouse did not only show a two-fold increase IL-6 levels, but this effect was moderated by the G allele of candidate variant rs1800795 in the *IL6* gene (64), the same variant implicated in the aforementioned candidate gene study on the association between *IL6* SNPs and suicide (45), and identified also in our analyses in two variant clumps. Similarly to the study of Schultze-Florey et al. in our models rs1800795 interacted with environmental influences, but with early childhood adversities rather than recent life events. Furthermore, in line with the results of Schultze-Florey et al. the C allele which was associated with an unchanged IL-6 level in the face of stress emerged as a protective allele in our models. Finally, we observed no main effect of this variant, similarly to the results of Schultze-Florey et al. where genotypes alone did not account for observed differences in circulating IL-6 in bereaved subjects and non-bereavers (64).

While this SNP did not show an effect in our study in interaction with recent stress, we found several clumps of variants interacting with negative life events occurring in the past year on current suicidal ideation. We could not identify clumps of variants interacting with recent stress on other suicide risk markers such as current thoughts of death or hopelessness. However, as specific genes and variants may mediate the effects of only specific types of stressors (53–55, 65), we also looked at the interaction between *IL6* variation and distinct recent stress types. Our models identified separate variant clumps significantly interacting with recent financial problems or personal problems on suicidal ideation, and with intimate relationship problems and social network problems on current thoughts of death, which once again suggests that different types of stressors may exert their effect *via* the mediation of different genetic variants.

### Effects of *IL6* Variation on Suicidal Behaviour in Interaction With Childhood Adversity

Early life adversity has been described as a major contributor to enhanced vulnerability to lifetime suicide (66). Childhood maltreatment affects immune response predisposing to heightened inflammatory states lasting into adult life with long-term consequences on both brain and behaviour (67), including abnormal cortisol stress responses, persistently increased levels of inflammatory cytokines including IL-6 and TNFα, low-grade elevations in other proinflammatory markers such as CRP, as well as greater inflammatory responses to later psychosocial stress (68). In a study investigating depressed patients raised inflammatory gene expression including cytokine-producing genes was linked to childhood adversity coupled with high suicide risk, and reduced gene expression was observed in those without childhood adversity and low suicide risk, suggesting the involvement of the immune system as a mediator between childhood adversity and increased suicide risk through a long-lasting activation of inflammation (69). In our study, variation in *IL6* showed robust interaction effects with childhood adversity on both lifetime suicide attempts and current suicidal ideation and current thoughts of death, emphasising the role of inflammation and as well as the role of *IL6* variation in predisposing to suicide risk in those exposed to early adversities, which might be exploited for risk screening and identification of those particularly in need of preventive interventions. As mentioned before, it is also notable that we identified in our clumps two variants previously investigated in association with suicide in candidate variant studies, providing further support to their putative role.

### Hopelessness Is Not Associated With Variation in the *IL6* Gene

Hopelessness has long been identified as one of the key and independent risk factor of suicidal behaviour (70). In our study we found no association between *IL6* variation and hopelessness, either as a main effect or in interaction with recent stress or childhood adversities. This is surprising in the light of previous studies which found positive correlations between IL-6 levels and hopelessness in serum and saliva samples taken at different times of the day (71), reported associations between hopelessness and IL-6 levels in depressed patients (72), and found that adolescents exhibiting higher levels of hopelessness reflecting cognitive vulnerability responded with higher increase of IL-6 levels to laboratory-based acute stress (73).

### Functional Prediction and Effects of Identified Variants in Previous Studies

While in our study we focused on the effects of linkage disequilibrium-based clumps of variants in the *IL6* gene rather than on single SNPs, and used top SNPs only to characterise the identified significant clumps, several of the member SNPs of significant clumps have previously been implicated in the literature in association with possibly relevant phenotypes.

Most importantly, as mentioned above, rs1800795, a member of two significant clumps interacting with childhood adversities on current thoughts of death, have previously been implicated in association with suicide (45), although with another direction; and with higher circulating IL-6 levels for G allele carriers in interaction with recent stress (64), which corresponds to our results where the minor C was associated with a protective effect. It is also noteworthy that in another study we have previously reported that the rs1800795 in interaction with recent stress increased depression risk and had an impact on depression severity (74). These convergent findings underscore the role of this particular variant in mediating the effects of stress on suicide and related phenotypes. rs1800795 has also been associated with a number of somatic conditions including cancer, inflammatory and autoimmune disorders. rs1800795 is located in the putative promoter of *IL6* and affects binding of transcription factors GATA1, GATA2, and YYI as well as NF1 and Sp1 (49) with likely functional consequences as reflected by the positive associations for this variant in divergent studies.

Rs7801617, a member SNP in a clump significantly interacting with childhood adversities on previous suicide attempts has previously been implicated in response to escitalopram in the GENDEP study (75). Another SNP in the same clump, rs2069824, has also been associated with treatment response in major depression and suggested to be a candidate for further investigation (76). These results may once again converge with our findings suggesting further studies on the association of these *IL6* variants with the efficacy of antidepressants in reducing the risk of suicidal behaviour.

Rs1880241, a top SNP of a clump significantly interacting with childhood adversities on lifetime suicide attempts has been found to be related to level of circulating CRP in a study revealing a protective effect of CRP on schizophrenia and a risk effect of CRP on bipolar disorder (77). IL-6 is an upstream stimulator of CRP production (35, 47), and many of its effects on relevant phenotypes may be mediated by CRP. Finally, rs4719714, top SNP of a clump significantly interacting with childhood adversities on current thoughts of death have been implicated in association with fatigue and sleep disturbances (78, 79).

Several of our identified variants have been implicated in other conditions such as somatic illnesses, references to these and results of functional prediction are shown in Supplementary Tables. However, the functional consequences or the effect on cytokine levels or function in case of the majority of these variants are unknown.

### Clinical Utility of the Involvement of *IL6* Variation in Suicidal Behaviour and Implications for Further Research

Understanding the role of inflammation and the contribution of the *IL6* gene has several potential clinical implications. *IL6* genotype may be utilised as a potential biomarker to identify specific subgroups of depressed patients with increased vulnerability for inflammation; and to detect at-risk subjects especially following early childhood adversities or recent stress and possibly target them with preventive measures focusing on inflammation (6). In this regard it is important that besides anti-inflammatory agents, psychological therapies, such as cognitive-behaviour therapy (80) or supportive-expressive dynamic psychotherapy (SEDP) has been shown to decrease IL-6 levels (81), without the side-effects of pharmacological agents, making subjects who carry the risk variants and exposed to stress suitable candidates for such intervention. The association of *IL6* genotype with the efficacy of such therapies on decreasing both IL-6 levels and suicide risk might also be tested. One further important question arising partly from our study is that whether childhood adversities in interaction with *IL6* genotype have a harmful effect on multiple measures of suicide risk, then how viable it is to offset or remediate the dire consequences of childhood trauma and maltreatment (67) before the onset of clinical symptoms by psychosocial or pharmacological interventions focusing on inflammation.

Besides finding potential biomarkers of acute and long-term suicide risk, a novel approach to treatment of suicide with novel treatment targets is also needed, especially as current treatments focus solely on treating the primary psychiatric diagnosis (5). Specific effects of cytokines on emotion and behaviour in different brain areas could be exploited for more optimal treatment targets, by identifying upstream triggers of inflammation and downstream neurobiological effectors and moderators conveying vulnerability or resilience (5). There is significant therapeutic promise in addressing different facets of the immune system (4), and immunotherapies may be helpful for depressed patients with low-grade inflammation or associated risk factors or suicide (47). Besides potential new targets, several anti-inflammatory treatments clinically approved for other indications might be tested in suicidal patients. Once more, use of potential biomarkers such as *IL6* variation may help identify those who may benefit from such agents even in the face of the abovementioned limiting factors.

### Limitations

Several limitations of our study must be taken into account when interpreting the impact of our results. First, lifetime suicide attempts, as well as both early childhood adversities and recent life events occurring in the past year were retrospectively assessed based on self-report, and are thus subject to recall and reporting biases. Current suicidal ideations, thoughts of death and hopelessness are similarly self-reported. Second, assessment of childhood adversity and recent negative life events does not take into consideration the differing severity and subjective impact of individual life events. Thirdly, we could not exclude the contribution of depression to our suicidal phenotypes. Finally, besides *IL6, IL6R* encoding the IL-6 receptor is also very important in determining IL-6 levels and functions, thus variation in this gene should also be covered by future broader studies to understand the impact of *IL6*. Nevertheless, our study also has several strengths, including consideration of several variants without hypothesis-based candidate SNP selection along the *IL6* gene with a linkage disequilibrium-based clumping method, employing different manifestations of suicidal behaviour along the suicide spectrum as phenotypes, and using a GxE paradigm with two etiologically different types of stressors.

## Conclusion

In conclusion, our study investigating the role of variation in the *IL6* gene with a linkage disequilibrium-based clumping approach identified several clumps of SNPs that influence both the risk of suicide attempts and current suicidal ideation and thoughts of death. Our results may bring us a step further in understanding the role of inflammation and specifically of IL-6 in suicidal behaviour, and identify novel biological markers of suicide risk especially in those exposed to stressful experiences, as well as to foster the adaptation of a new paradigm and identify novel approaches and targets in the treatment of suicidal behaviour.

## Data Availability Statement

The datasets presented in this study can be found online in the FigShare repository: https://figshare.com/s/6d0f6f781466c78a7c0a.

## Ethics Statement

The studies involving human participants were reviewed and approved by North Manchester Local Research Ethics Committee; and Scientific and Research Ethics Committee of the Medical Research Council, Budapest, Hungary. The patients/participants provided their written informed consent to participate in this study.

## Author Contributions

XG, JB, GB, and GJ designed and conceptualised the study and collected the data. SS, DG, NE, and ZG participated in statistical analyses. DT carried out *in silico* functional analyses. XG, JB, and DT wrote the first draught of the manuscript. SS and ZG created figures. All authors participated in interpreting the data, developing further and final versions of manuscript, and contributed and have approved the final manuscript.

## Funding

This study was supported by the Sixth Framework Program of the European Union (NewMood, LSHM-CT-2004-503474), the Hungarian Academy of Sciences (MTA-SE Neuropsychopharmacology and Neurochemistry Research Group), the Hungarian Brain Research Program (Grants: 2017-1.2.1-NKP-2017-00002 and KTIA_13_NAPA-II/14), the National Development Agency (Grant: KTIA_NAP_13-1-2013- 0001), the Hungarian Academy of Sciences, Hungarian National Development Agency, Semmelweis University and the Hungarian Brain Research Program (Grant: KTIA_NAP_13-2- 2015-0001) (MTA-SE-NAP B Genetic Brain Imaging Migraine Research Group), the ITM/NKFIH Thematic Excellence Programme, Semmelweis University; the National Research, Development and Innovation Office, Hungary (2019-2.1.7-ERA-NET-2020-00005), under the frame of ERA PerMed (ERAPERMED2019-108), and the Thematic Excellence Programme (Tématerületi Kiválósági Program, 2020-4.1.1.-TKP2020) of the Ministry for Innovation and Technology in Hungary, within the framework of the Neurology and Translational Biotechnology thematic programmes of the Semmelweis University; and the SE-Neurology FIKP grant of EMMI. SS was supported by ÚNKP-19-1-1-PPKE-63 New National Excellence Program of the Ministry for Innovation and Technology. NE was supported by the New National Excellence Program of The Ministry for Innovation and Technology from the source of the National Research, Development and Innovation Fund (ÚNKP-20-4-II-SE-9). DB was supported by the New National Excellence Program of the Ministry for Innovation and Technology from the source of the National Research, Development and Innovation Fund (ÚNKP-20-3-II-SE-51). The sponsors had no further role in the study design; in the collection, analysis and interpretation of data; in the writing of the report; and in the decision to submit the paper for publication.


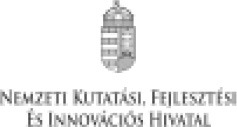
 
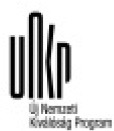


## Conflict of Interest

The authors declare that the research was conducted in the absence of any commercial or financial relationships that could be construed as a potential conflict of interest.

## Publisher's Note

All claims expressed in this article are solely those of the authors and do not necessarily represent those of their affiliated organizations, or those of the publisher, the editors and the reviewers. Any product that may be evaluated in this article, or claim that may be made by its manufacturer, is not guaranteed or endorsed by the publisher.

## Supplementary Material

Supplementary Table 1 Individual main effects of the investigated *IL6* variants on lifetime suicide attempts (SUIC), current suicidal ideation (SI-BSI03), hopelessness (H-BSI18), and current thoughts of death (ToD-BSI21).

Supplementary Table 2 Individual interaction effects of the investigated *IL6* variants and childhood adversities (CHA) on lifetime suicide attempts (SUIC), current suicidal ideation (SI-BSI03), hopelessness (H-BSI18), and current thoughts of death (ToD-BSI21).

Supplementary Table 3 Individual interaction effects of the investigated *IL6* variants and recent life events (RLE) on current suicidal ideation (SI-BSI03), hopelessness (H-BSI18), and current thoughts of death (ToD-BSI21).

Supplementary Table 4 Functional prediction results for SNPs in the significant clump emerging in interaction with childhood adversities (xCHA) on lifetime suicide attempts (SUIC) in the additive model.

Supplementary Table 5 Functional prediction results for SNPs in the two significant clumps emerging in interaction with childhood adversities (xCHA) on lifetime suicide attempts (SUIC) in the dominant model.

Supplementary Table 6 Functional prediction results for SNPs in the two significant clumps emerging in interaction with childhood adversities (xCHA) on current suicidal ideation (SI-BSI03) in the additive model.

Supplementary Table 7 Functional prediction results for SNPs in the significant clump emerging in interaction with childhood adversities (xCHA) on current suicidal ideation (SI-BSI03) in the dominant model.

Supplementary Table 8 Functional prediction results for SNPs in the significant clump emerging in interaction with childhood adversities (xCHA) on current thoughts of death (ToD-BSI21) in the additive model.

Supplementary Table 9 Functional prediction results for SNPs in the significant clump emerging in interaction with childhood adversities (xCHA) on current thoughts of death (ToD-BSI21) in the dominant model.

Supplementary Table 10 Functional prediction results for SNPs in the significant clump emerging in interaction with childhood adversities (xCHA) on current thoughts of death (ToD-BSI21) in the recessive model.

Supplementary Table 11 Functional prediction results for SNPs in the significant clump emerging in interaction with recent life events (xRLE) on current suicidal ideation (SI-BSI03) in the dominant model.

Supplementary Table 12 Functional prediction results for SNPs in the two significant clumps emerging in interaction with recent life events (xRLE) on current suicidal ideation (SI-BSI03) in the recessive model.
